# TMT-based quantitative proteomics analysis of serum-derived exosomes in patients with juvenile gout

**DOI:** 10.3389/fendo.2025.1460218

**Published:** 2025-05-15

**Authors:** Zhuyi Ji, Shaoling Zheng, Ling Liang, Lixin Huang, Shanmiao Sun, Zhixiang Huang, Yuebing He, Xia Pan, Tianwang Li, Yukai Huang

**Affiliations:** ^1^ The Affiliated Guangdong Second Provincial General Hospital of Jinan University, Guangzhou, China; ^2^ Department of Rheumatology and Immunology, Zhaoqing Central People’s Hospital, Zhaoqing, China; ^3^ Department of Rheumatology and Immunology, The Third People’s Hospital of Chengdu, Chengdu, China; ^4^ The Second Affiliated Hospital, Guangzhou Medical University, Guangzhou, China

**Keywords:** juvenile gout, exosomes, TMT, proteomics, biomarker

## Abstract

**Objectives:**

The purpose of this study was to compare the proteomics of serum-derived exosomes in juvenile gout (J-Gout), juvenile hyperuricemia (J-HUA) and oligoarticular juvenile idiopathic arthritis (oJIA).

**Methods:**

Serum-derived exosomes were isolated from patients using a qEV column combined with the ExoQuick-TC kit. The proteomics of serum-derived exosomes was analyzed by tandem mass tag (TMT)-labeled liquid chromatography-mass spectrometry (LC-MS/MS) technology. Proteins differentially expressed in J-Gout and the other two groups were identified. This was followed by volcano plot, hierarchical cluster, Venn diagram, gene ontology (GO), and Kyoto Encyclopedia of Genes and Genome (KEGG) pathway analyses.

**Results:**

A total of 838 credible proteins were identified in serum-derived exosomes from the three groups. Eighty-eight differentially expressed proteins (13 upregulated and 75 downregulated) were identified in J-Gout when compared with J-HUA. One hundred twenty-one differentially expressed proteins (20 upregulated and 101 downregulated) were identified in J-Gout when compared with oJIA. A total of 166 differentially expressed proteins were identified in J-Gout, compared with J-HUA and oJIA respectively. Bioinformatic analysis indicated that the 166 differentially expressed proteins were significantly involved in “immune response”, “Fc epsilon RI signaling pathway” and “B cell receptor signaling pathway”. A total of 43 differentially expressed proteins were identified in J-Gout, compared with J-HUA and oJIA simultaneously. Six proteins were found highly expressed in J-Gout uniquely. ELISA results showed that dipeptidyl peptidase 4 (DPP4) and heparin cofactor 2 (SERPIND1) were the highest in J-Gout, which was consistent with the proteomic results. Correlation analysis revealed that exosome-derived DPP4 and SERPIND1 were positively correlated with C-reactive protein (CRP) and erythrocyte sedimentation rate (ESR).

**Conclusion:**

The protein composition of serum-derived exosomes in J-Gout was significantly differed from that in J-HUA and oJIA. DPP4 and SERPIND1 were uniquely highly expressed in J-Gout. Some possible mechanisms regarding the inflammatory response and coagulation complement system were proposed, which may provide helpful diagnostic and therapeutic insights for J-Gout.

## 1 Introduction

Gout is an inflammatory form of arthritis that can be attributed to monosodium urate (MSU) deposition resulting from hyperuricemia (1). Recent data suggest that the incidence and prevalence of juvenile gout (J-Gout) are increasing, and childhood obesity parallels the increased incidence of gout at younger ages (2, 3). A survey based on Chinese children and adolescents showed an overall prevalence of hyperuricemia of 23.3% (4). At present, few studies exploring J-Gout have been conducted, and many challenges still exist in J-Gout diagnosis and treatment. A previous study showed that compared with adult gout, J-Gout has a higher average level of serum uric acid and faster progression of joint destruction (5). In addition, most J-Gout patients meet the diagnostic criteria for juvenile idiopathic arthritis (JIA), especially oligoarticular juvenile idiopathic arthritis (oJIA) (6). Therefore, it is of vital significance to find biomarkers for J-Gout and further explore its pathogenesis.

Exosomes are nanoscale membrane vesicles with a diameter of 30–150 nm that contain proteins and RNAs and are present in serum, synovial fluid, urine, and milk. Exosomes are thought to be an essential mediators of intercellular communication and carriers of cargoes involved in cellular processes, including extracellular matrix degradation, inflammation regulation, and antigen presentation (7–9). Yoo identified that exosomal serum amyloid A (SAA) and lymphatic endothelial hyaluronic acid receptor-1 (LYVE-1) were important in the rheumatoid arthritis (RA) pathogenic process and could serve as novel biomarkers of activity and remission (10). Ying screened and identified differentially expressed proteins using proteomics and found that the TBB4A protein may be involved in the pathogenesis of gout (11). Li analyzed the protein profiles of synovial fluid-derived exosomes from adult gout patients and proposed some potential biomarkers (12). However, no study has been conducted examining the proteomics of serum-derived exosomes in J-Gout.

In our study, serum-derived exosomes were isolated from J-Gout, juvenile hyperuricemia (J-HUA) and oJIA patients. Quantitative proteomics with tandem mass tag (TMT) labeling combined with LC-MS/MS was used to explore differentially expressed proteins. This study may provide clues for identifying potential biomarkers and further exploring the molecular mechanism of J-Gout.

## 2 Materials and methods

### 2.1 Participants

Because J-Gout and J-HUA do not have official acronyms, we customized the study subjects for the experiment. Gout and hyperuricemia were diagnosed according to adult criteria, but all subjects were aged <18 years.

In this study, a total of 6 J-Gout patients with acute attacks, 6 J-HUA patients and 6 oJIA patients from October 2018 to August 2022 in our hospital were enrolled. The inclusion criteria were as follows: all cases were aged <18 years. Gout was diagnosed in accordance with the 2015 American College of Rheumatology/European League Against Rheumatism classification criteria for primary gout (13). The diagnosis of HUA was consistent with serum uric acid (sUA) levels greater than 420 μmol/L for boys and 360 μmol/L for girls, no history of acute attack gout and no medical treatment (14). OJIA was diagnosed according to the International League of Associations for Rheumatology (ILAR) criteria (15). The serum was centrifuged, and the supernatant was stored at −80°C. Samples were collected after obtaining informed consent from all participants. This study was approved by the Ethics Committee of the Guangdong Second Provincial General Hospital (2019-QNJJ-17-02).

### 2.2 Isolation and identification of exosomes

Serum-derived exosomes were isolated from patients using a qEV column combined with the ExoQuick-TC kit. The morphology of exosomes was observed by transmission electron microscopy (TEM). The size and concentration of exosomes were measured by high-sensitivity flow cytometry (HSFC) for nanoparticle analysis. Western blotting was used to examine the levels of exosome protein markers (TSG101 and CD81).

### 2.3 Tryptic digestion

Six samples in the same group were mixed pairwise into three samples to be tested per group. Corresponding volumes of 25 mM dithiothreitol and 100 mM iodoacetamide were added. After incubating away from light, acetone was added (6 times volume) to precipitate the protein. After being left overnight, the precipitate was collected by centrifugation at 8000 g for 10 min, and 200 mM tetraethylammonium bromide was added to bring the volume to 100 µl. The samples were digested with trypsin overnight at 37°C in a 50:1 ratio (protein: enzyme), followed by lyophilization.

### 2.4 TMT labeling

The lyophilized samples were added to 100 mM tetraethylammonium bromide, followed by the addition of TMT pro reagent mixed with anhydrous acetonitrile. After leaving for 1 h, 5% hydroxylamine was added to react for 15 min. The labeled peptide solutions were lyophilized.

### 2.5 Reversed-phase chromatography separation

The samples were fractionated by reversed-phase 1100 HPLC using an Agilent Zorbax Extend-C18 narrow diameter column (2.1×150 mm, 5µm, Agilent, USA). The detection wavelengths were set to 210 nm and 280 nm. The flow rate was set to 300 µL/min, mobile phase A (2% acetonitrile in HPLC water) and mobile phase B (90% acetonitrile in HPLC water). Samples were collected by gradient elution for 8–60 min, and the eluate was collected in centrifuge tubes every minute. The centrifuge tubes were marked 1-15, and samples were repeatedly collected in these tubes. The separated samples were lyophilized for mass spectrometry analysis.

### 2.6 Chromatography and mass spectrometry conditions

The samples were loaded onto the precolumn Acclaim PepMap100 (Thermo, USA), setting at a flow rate of 350 nL/min and separated using an Acclaim PepMap RSLC (RP-C18, Thermo Fisher, USA) separation column. Full MS scans were acquired in the mass range of 350–1500 m/z with a mass resolution of 60,000, an AGC target of 3e6 and a maximum injection time of 50 ms. MS/MS spectra were obtained with a resolution of 45,000, an AGC target of 2e5 and a maximum injection time of 80 ms. All MS/MS spectra were obtained in positive ion mode, and the dynamic exclusion time was set to 30 s.

### 2.7 Database search

The resulting MS/MS data were processed using Proteome Discoverer 2.4.1.15 (Thermo Fisher Scientific, USA). Trypsin was specified as a cleavage enzyme allowing up to 2 missing cleavages. The primary MS error range was 10 ppm, and the fragment ion mass tolerance was 0.02 Da.

### 2.8 Bioinformatics analysis

Differential proteins were screened according to the criteria of fold change ≥ 1.2 and *p* value < 0.05 and analyzed by volcano plot, hierarchical clustering heatmap and Venn diagram. The functions of the differentially expressed proteins were assessed by GO enrichment analysis, which comprehensively describes the functions of genes and products in organisms in terms of biological processes, cellular components, and molecular functions. The Kyoto Encyclopedia of Genes and Genomes (KEGG) (http://www.genome.jp/kegg/ and https://david.ncifcrf.gov/) was used to analyze the biological regulatory pathways and functional roles of proteins with significantly differential expression.

### 2.9 ELISA

Protein samples of serum-derived exosomes from 6 J-Gout, 6 J-HUA and 6 oJIA patients were measured for the expression levels of DPP4 and SERPIND1 using ELISA kits (ZCIBIO-32912, ZCIBIO-56060) according to the manufacturer’s instructions.

### 2.10 Statistical analysis

All statistical analyses were conducted using SPSS 23.0 or GraphPad Prism 8 software. Continuous variables are described as the mean ± standard deviation (mean ± SD) in the patient’s basic information, and categorical variables are described as frequencies. One-way ANOVA or nonparametric tests were used for continuous variables, and differences between groups were assessed using categorical variables and chi-square tests or Fisher’s exact probability method. Bivariate correlation analysis was performed using Pearson correlation analysis. A p value < 0.05 was accepted as statistically significant.

## 3 Results

### 3.1 Clinical characteristics of the participants

J-Gout patients were older than oJIA patients. J-Gout patients had higher levels of white blood cell counts than J-HUA patients. Hemoglobin levels were higher in J-Gout patients than in oJIA patients. J-Gout patients had the highest sUA levels compared to oJIA and J-HUA patients. All differences were statistically significant (*p* < 0.05) (**Table 1**).

**Table 1:** Basic characteristics of the participants.

	J-Gout (n=6)	J-HUA (n=6)	oJIA (n=6)	*p* value
Age (years)	14.33 ± 1.63	12.17 ± 3.97	6.00 ± 3.95*	0.002
Sex (male/female)	6/0	5/1	2/4	0.027
WBC (10^9/mL)	8.36 ± 1.12	5.42 ± 1.81*	10.30 ± 2.60	0.004
NE# (10^9/mL)	4.84 ± 0.97	3.16 ± 1.32	5.83 ± 1.80	0.024
PLT (10^9/mL)	307.67 ± 59.95	232.00 ± 69.15	386.83 ± 68.68	0.006
HGB (g/L)	152.33 ± 6.15	140.00 ± 14.54	124.67 ± 8.57*	0.001
CRP (mg/L)	14.02 ± 27.60	0.78 ± 1.56	20.58 ± 21.65	0.394
ESR (mm/h)	24.52 ± 25.03	5.28 ± 4.29	47.20 ± 26.75	0.029
sUA (umol/L)	606.17 ± 132.52	443 ± 92.46*	273.50 ± 36.23*	<0.001
RF (IU/mL)	2.04 ± 2.81	4.2 ± 3.54	3.95 ± 1.55	0.388
CCP (U/mL)	10.72 ± 3.03	18.27 ± 2.73	15.47 ± 12.76	0.492

*p < 0.05 vs. J-Gout group.

### 3.2 Isolation and identification of serum-derived exosomes

**Figure 1:**
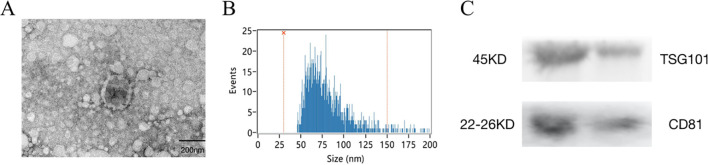
Isolation and identification of serum-derived exosomes. **(A)** The morphology of exosomes was shown by transmission electron microscopy (TEM), Scale bar=200 nm. **(B)** The size of exosomes was detected by high -sensitivity flow cytometry (HSFC) nanoparticle analysis. **(C)** The expression of CD81 and TSG101 was detected by western blotting.

Morphological analysis using TEM showed that exosomes were round to oval vesicular structures with darker stained lipid bilayers and lighter stained low electron density material (**Figure 1A**). The HSFC nanoparticle analysis indicated that the exosome diameters were 78.69 ± 21.41 nm, and their concentration was 256 x 10^10^ particles/ml (**Figure 1B**). Western blot results showed that CD81 and TSG101 were significantly expressed (**Figure 1C**). The results indicated that the isolated exosomes had further experimental feasibility.

### 3.3 Quality control of proteomics data

**Figure 2:**
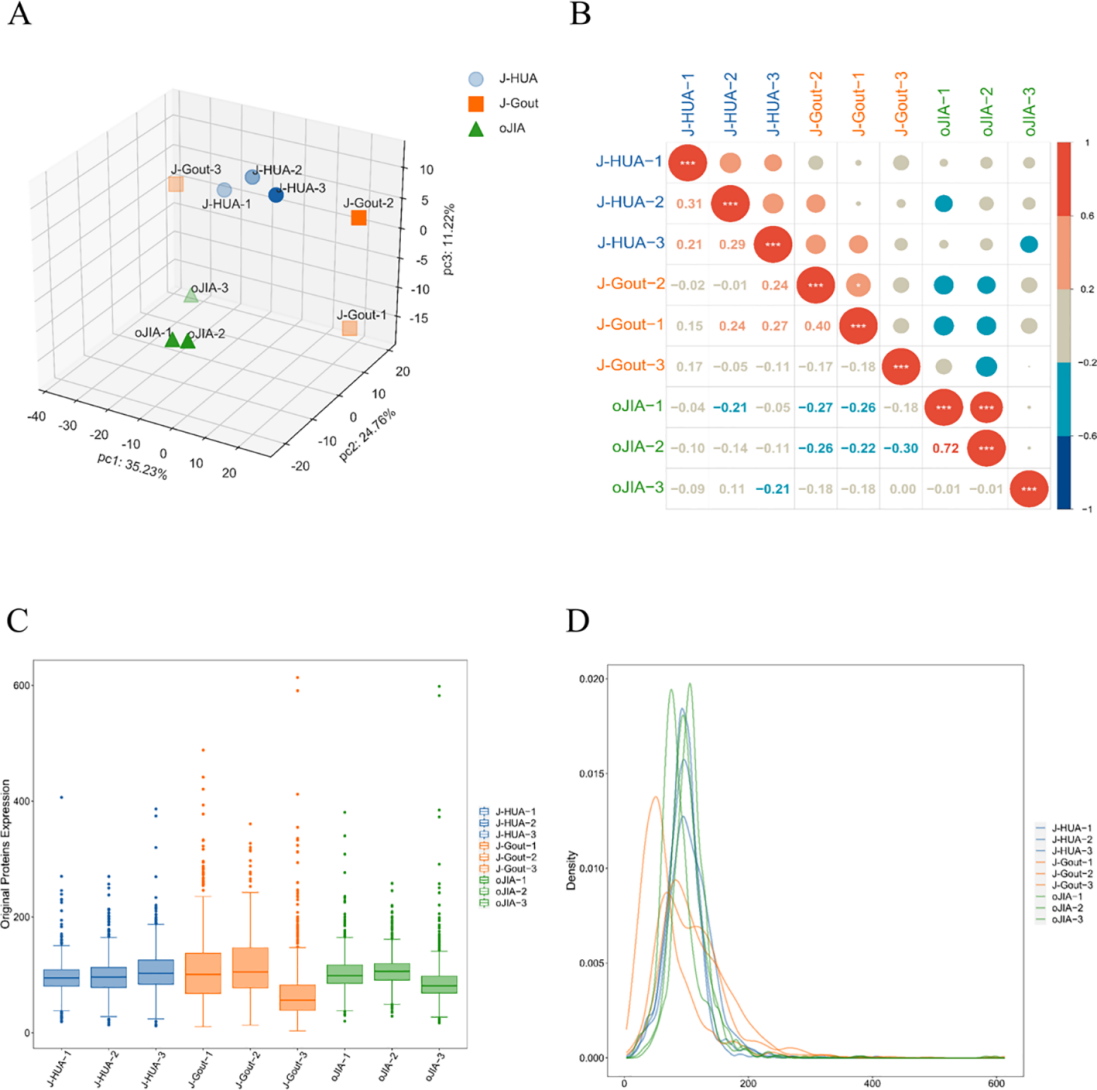
Quality control of proteomics data. **(A)** PCA. **(B)** Corrplot analysis. **(C)** Box plot analysis. **(D)** Density map analysis.

Principal component analysis (PCA) revealed differences between samples from different dimensions, and the results showed that the protein expression profiles of samples in the same group were basically stable (**Figure 2A**). Corrplot analysis showed that samples from the same group were strongly correlated (**Figure 2B**). Box and density plot analyses of credible protein expression revealed small fluctuations across samples and concentrations (**Figures 2C, D**). These results indicated sample stability and reproducibility.

### 3.4 Screening and functional analysis of differentially expressed proteins

A total of 838 credible proteins were identified in serum-derived exosomes from the three groups. Eighty-eight differentially expressed proteins were identified in J-Gout when compared with J-HUA. One hundred twenty-one differentially expressed proteins were identified in J-Gout when compared with oJIA. A total of 166 differentially expressed proteins were identified in J-Gout, compared with J-HUA and oJIA respectively. A total of 43 differentially expressed proteins were identified in J-Gout, compared with J-HUA and oJIA simultaneously. Six proteins were found highly expressed in J-Gout uniquely. All screens were based on criteria of log2 | fold change | ≥ 1.2 and p < 0.05. The Kyoto Encyclopedia of Genes and Genomes (KEGG) (http://www.genome.jp/kegg/ and https://david.ncifcrf.gov/) was used to analyze the biological regulatory pathways and functional roles of proteins with significantly differential expression.

#### 3.4.1 Screening and functional analysis of differentially expressed proteins in J-Gout vs. J-HUA

**Figure 3:**
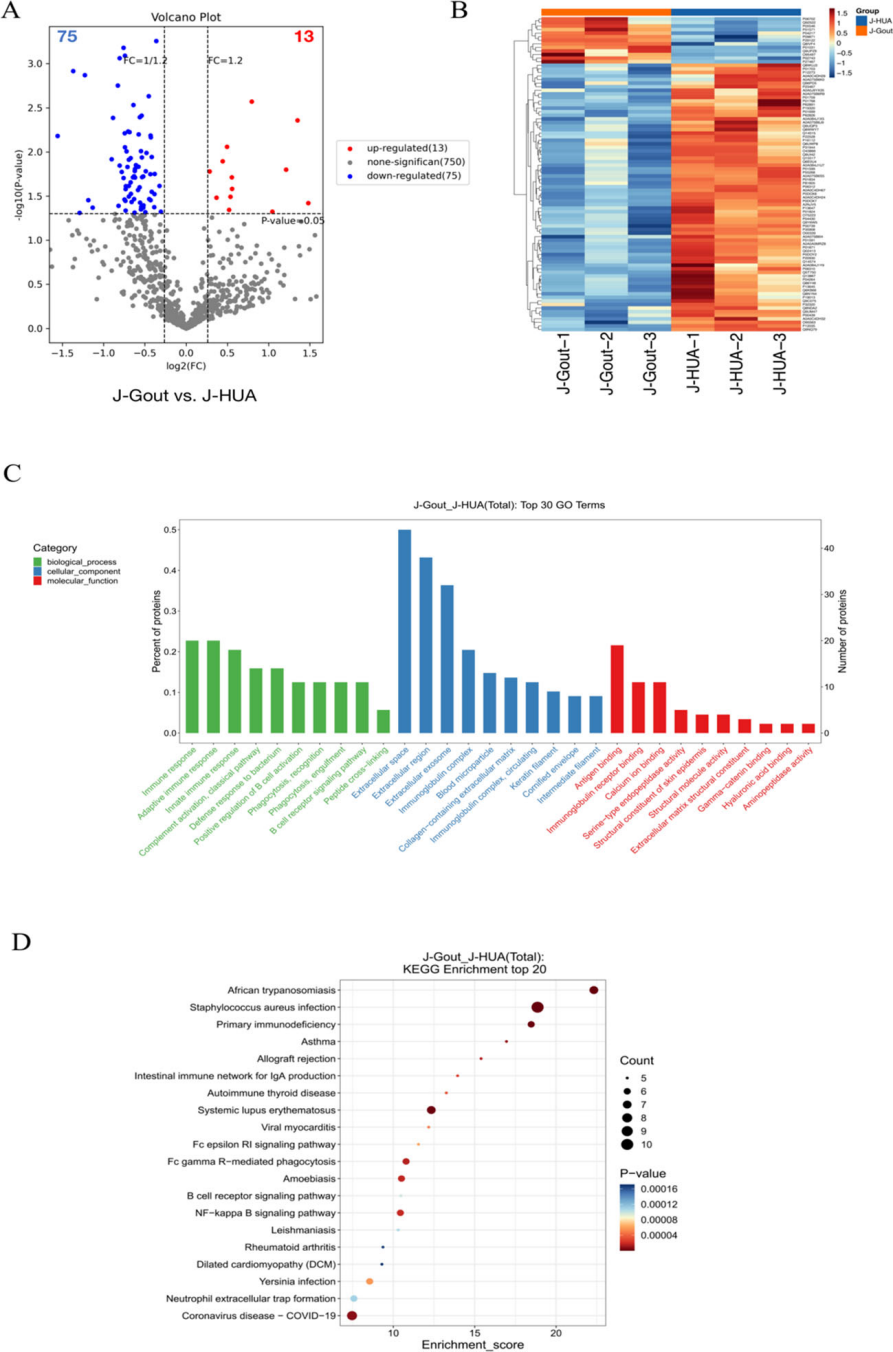
Screening and functional analysis of differentially expressed proteins in J-Gout vs. J-HUA. **(A)** Volcano plots. **(B)** Hierarchical clustering analysis. In the color bar, red represents upregulated expression, and blue represents downregulated expression. **(C)** GO analysis. **(D)** KEGG pathway analysis.

Proteins that were differentially expressed in J-Gout and J-HUA groups were identified according to the criteria of log2 | fold-change | ≥ 1.2 and *p* < 0.05. The volcano plot results showed that compared with the J-HUA, 13 and 75 proteins were upregulated and downregulated in J-Gout, respectively (**Figure 3A**). Upregulated proteins included histone H1.10 (H1-10) and heparin cofactor 2 (SERPIND1), and downregulated proteins included immunoglobulin kappa variable 1-17 (IGKV1-17) and immunoglobulin kappa variable 6-21 (IGKV6-21) (**Table 2**). Hierarchical clustering analysis was performed to reveal the dynamic profiles of differentially expressed proteins in the two groups (**Figure 3B**). Bioinformatic analysis indicated that the differentially expressed proteins were significantly involved in “immune response”, “Fc epsilon RI signaling pathway”, “B cell receptor signaling pathway” and “neutrophil extracellular trap formation” (**Figures 3C, D**).

**Table 2:** The differentially expressed proteins in J-Gout vs. J-HUA.

Accession number	Gene name	Description	J-Gout/J-HUA FC	*P* value
Q92522	H1-10	Histone H1.10	2.788711474	0.038
P05546	SERPIND1	Heparin cofactor 2	2.545981773	0.004
Q9UPZ9	CILK1	Serine/threonine-protein kinase ICK	2.31308222	0.016
O95497	VNN1	Pantetheinase	2.062615101	0.047
P01031	C5	Complement C5	1.733464956	0.003
P01599	IGKV1-17	Immunoglobulin kappa variable 1-17	0.339959225	0.007
A0A0C4DH24	IGKV6-21	Immunoglobulin kappa variable 6-21	0.386996904	0.001
A0A075B6K0	IGLV3-16	Immunoglobulin lambda variable 3-16	0.408347928	0.049
P12035	KRT3	Keratin, type II cytoskeletal 3	0.426857143	0.001
P16112	ACAN	Aggrecan core protein	0.43933518	0.035

Gray represents upregulated proteins, and white represents downregulated proteins.

#### 3.4.2 Screening and functional analysis of differentially expressed proteins in J-Gout vs. oJIA

**Figure 4:**
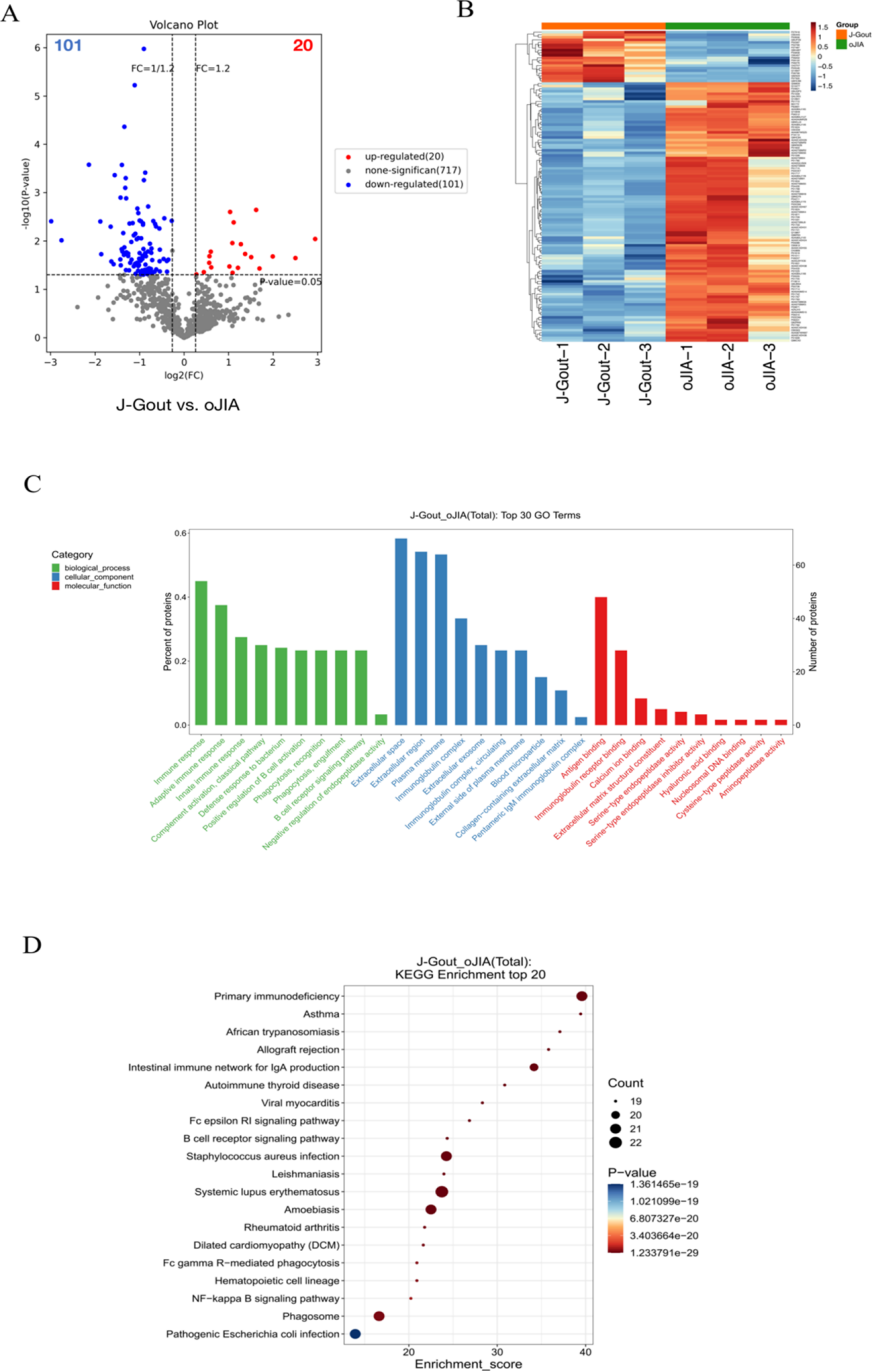
Screening and functional analysis of differentially expressed proteins in J-Gout vs. oJIA. **(A)** Volcano plots. **(B)** Hierarchical clustering analysis. In the color bar, red represents upregulated expression, and blue represents downregulated expression. **(C)** GO analysis. **(D)** KEGG pathway analysis.

Proteins that were differentially expressed in J-Gout and oJIA groups were identified according to the criteria of log2 | fold-change | ≥ 1.2 and *p* < 0.05. The volcano plot results showed that compared with the oJIA, 20 proteins were upregulated, while 101 were downregulated in J-Gout (**Figure 4A**). Upregulated proteins included histone H1–10 and spondin-1 (SPON1), and downregulated proteins included immunoglobulin lambda variable 3-25 (IGLV3-25) and immunoglobulin lambda variable 3-1 (IGLV3-1) (**Table 3**). Hierarchical clustering analysis was performed to reveal the dynamic profiles of differentially expressed proteins in the two groups (**Figure 4B**). Bioinformatic analysis indicated that the differentially expressed proteins were significantly involved in “immune response”, “Fc epsilon RI signaling pathway”, “B cell receptor signaling pathway” and “primary immunodeficiency” (**Figures 4C, D**).

**Table 3:** Differentially expressed proteins in J-Gout vs. oJIA.

Accession number	Gene name	Description	J-Gout/oJIA FC	*P* value
Q92522	H1-10	Histone H1.10	7.694672131	0.009
Q9HCB6	SPON1	Spondin-1	5.661495063	0.022
P07305	H1-0	Histone H1.0	3.97469459	0.021
P28799	GRN	Progranulin	3.231152993	0.037
P05546	SERPIND1	Heparin cofactor 2	3.071464268	0.002
P01717	IGLV3-25	Immunoglobulin lambda variable 3-25	0.126243958	0.004
P01715	IGLV3-1	Immunoglobulin lambda variable 3-1	0.14797546	0.010
A0A0A0MS15	IGHV3-49	Immunoglobulin heavy variable 3-49	0.227100271	0.000
P01763	IGHV3-48	Immunoglobulin heavy variable 3-48	0.271468144	0.004
A0A075B6I4	IGLV10-54	Immunoglobulin lambda variable 10-54	0.275268817	0.019

Gray represents upregulated proteins, and white represents downregulated proteins.

#### 3.4.3 Screening and functional analysis of differentially expressed proteins in J-Gout vs. J-HUA and J-Gout vs. oJIA

**Figure 5:**
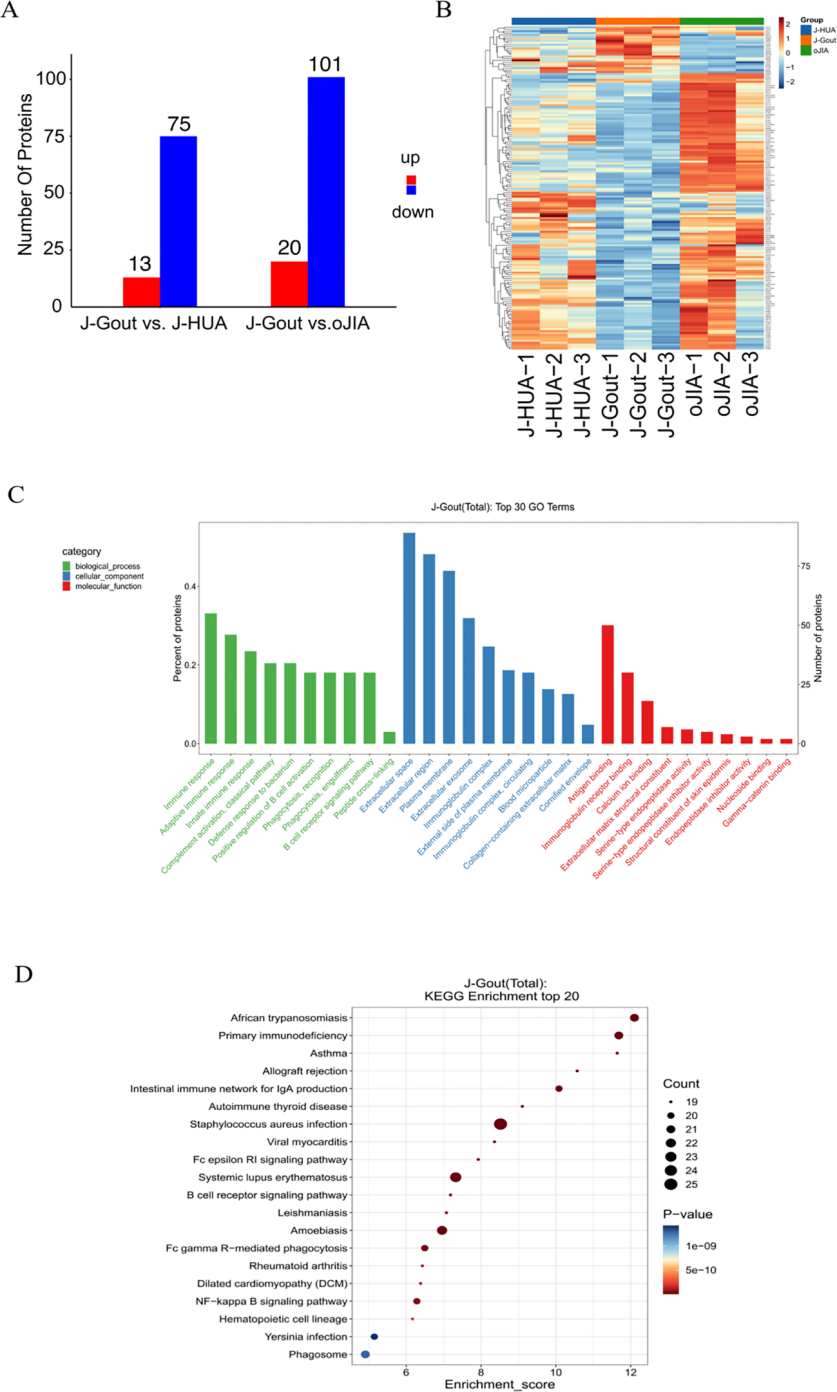
Screening and functional analysis of differentially expressed proteins in J-Gout vs. J-HUA and J-Gout vs. oJIA. **(A)** The number of differentially expressed proteins (J-Gout vs. J-HUA, J-Gout vs. oJIA). **(B)** Hierarchical clustering analysis of the three groups. In the color bar, red represents upregulated expression, and blue represents downregulated expression. **(C)** GO analysis. **(D)** KEGG pathway analysis.

A total of 166 differentially expressed proteins were identified when examining the combination of J-Gout vs. J-HUA and J-Gout vs. oJIA according to the criteria of log2 | fold-change | ≥ 1.2 and *p* < 0.05 (**Figure 5A**). Upregulated expression proteins included SPON1 and H1-10, and downregulated expression proteins included immunoglobulin lambda variable 10-54 (IGLV10-54) and IGKV1-17 (**Table 4**). Hierarchical clustering analysis was performed to reveal the dynamic profiles of differentially expressed proteins in the three groups (**Figure 5B**). Bioinformatic analysis indicated that the differentially expressed proteins were significantly involved in “immune response”, “Fc epsilon RI signaling pathway”, “B cell receptor signaling pathway”, “NF-kappa B signaling pathway” and “primary immunodeficiency” (**Figures 5C, D**).

**Table 4:** Differentially expressed proteins in J-Gout vs. J-HUA and J-Gout vs. oJIA.

Accession number	Gene name	Description	J-Gout/J-HUA FC	J-Gout/oJIA FC
Q9HCB6	SPON1	Spondin-1	2.626963351	5.661495063
Q92522	H1-10	Histone H1.10	2.788711474	7.694672131
P28799	GRN	Progranulin	1.78803681	3.231152993
Q9UPZ9	CILK1	Serine/threonine-protein kinase ICK	2.31308222	2.587675578
P05546	SERPIND1	Heparin cofactor 2	2.545981773	3.071464268
A0A075B6I4	IGLV10-54	Immunoglobulin lambda variable 10-54	0.456327986	0.275268817
P01599	IGKV1-17	Immunoglobulin kappa variable 1-17	0.339959225	0.403386755
A0A0C4DH24	IGKV6-21	Immunoglobulin kappa variable 6-21	0.386996904	0.439859245
A0A0B4J1X5	IGHV3-74	Immunoglobulin heavy variable 3-74	0.617647059	0.378823529
P16112	ACAN	Aggrecan core protein	0.43933518	0.418690602

Gray represents upregulated proteins, and white represents downregulated proteins.

#### 3.4.4 Screening of differentially expressed proteins in the intersection between J-Gout vs. J-HUA and J-Gout vs. oJIA

**Figure 6:**
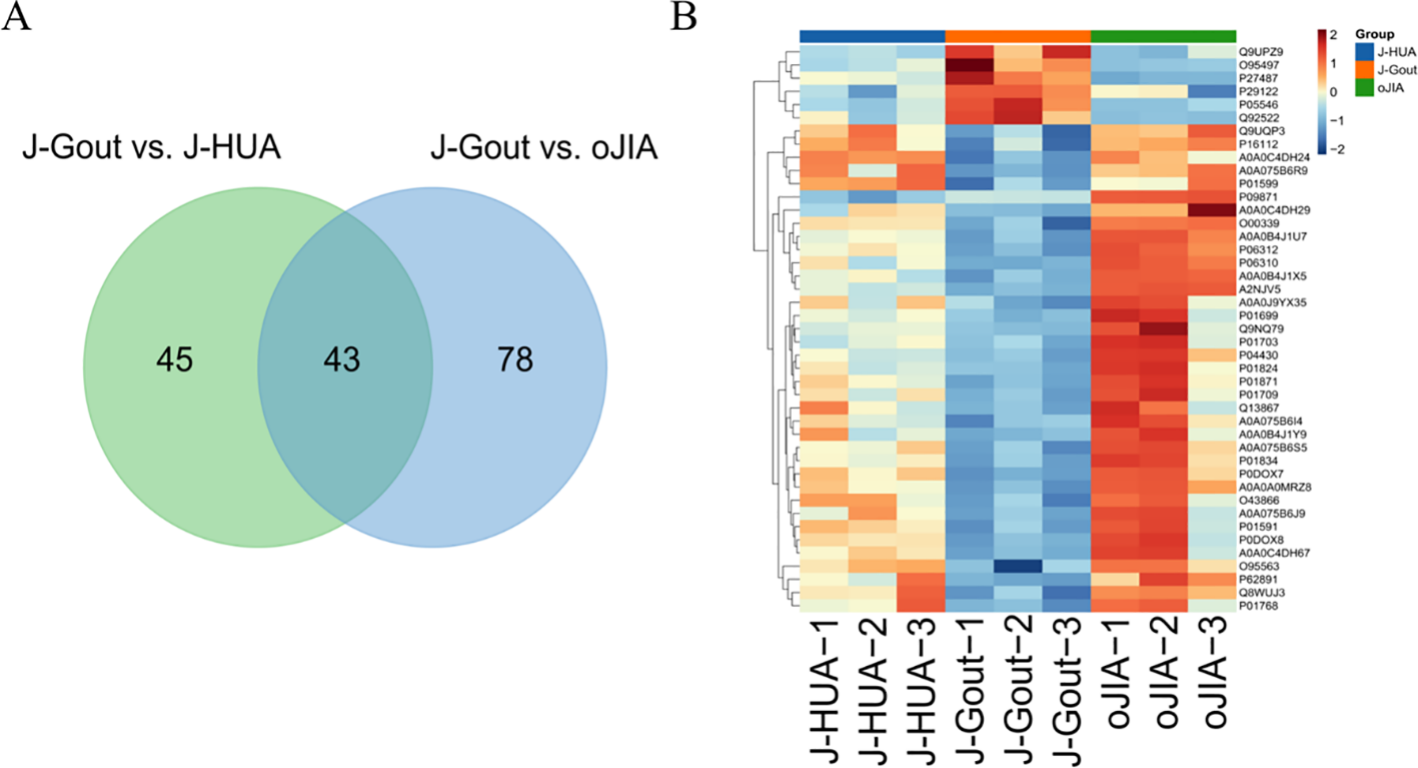
Screening of differentially expressed proteins in the intersection between J-Gout vs. J-HUA and J-Gout vs. oJIA. **(A)** Venn diagram analysis of J-Gout vs. J-HUA and J-Gout vs. oJIA. **(B)** Hierarchical clustering of the three groups.

A total of 43 differentially expressed proteins were identified in J-Gout based on the intersection of J-Gout vs. J-HUA and J-Gout vs. oJIA (**Figure 6A**). The cluster heatmap shows the most highly expressed proteins as red and the proteins expressed in low levels in J-Gout as blue (**Figure 6B**). With the criteria of ≥ 2 unique peptides, 6 proteins were found to be uniquely highly expressed in J-Gout, including H1-10, CILK1, SERPIND1, pantetheinase (VNN1), dipeptidyl peptidase 4 (DPP4), and proprotein convertase subtilisin/kexin type 6 (PCSK6) (**Table 5**).

**Table 5:** Proteins uniquely highly expressed in J-Gout.

Protein name	Abundance								
	J-Gout			J-HUA			oJIA		
H1-10	275.9	314	161.1	130.4	42.2	96.7	30.4	36.9	30.3
CILK1	211.9	137.6	221.6	83.1	89.2	74.6	63.3	54.3	103.1
SERPIND1	208.2	235.9	170.5	79.6	63.6	98.2	60.4	60.6	79.1
VNN1	200.9	114.1	133	62.6	69.4	85.2	49.6	52.8	55.5
DPP4	145.1	118.2	107.6	87.9	85.4	79.1	54	58.5	58.7
PCSK6	137.4	137.8	128.2	99.4	80.8	106	110.2	111.2	75

The above experimental results indicate that based on proteomic analysis, we screened significant differentially expressed proteins in juvenile gout.

### 3.5 Verification of DPP4 and SERPIND1 concentrations and correlation analysis with clinical indicators

**Figure 7:**
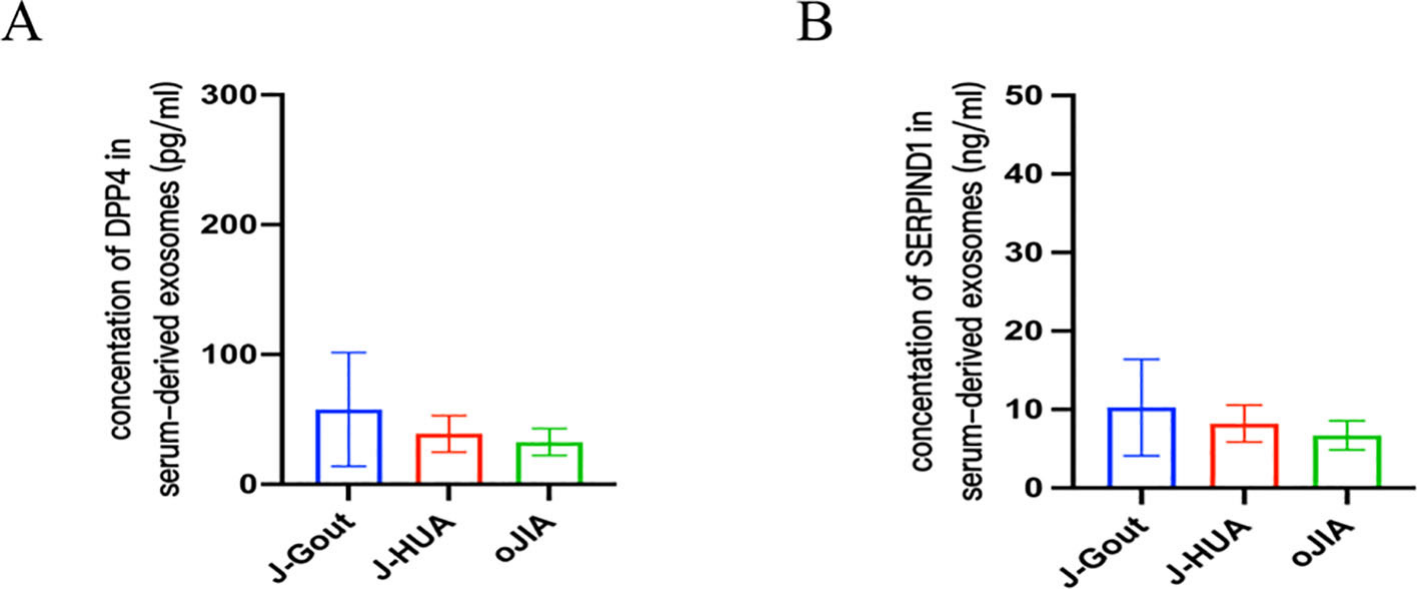
Verification of DPP4 and SERPIND1 protein expression by ELISA. **(A)** The level of DPP4 in serum-derived exosomes. **(B)** The level of SERPIND1 in serum-derived exosomes. (**A, B**, bars = mean ± standard error).

ELISA results showed that the concentrations of DPP4 in serum-derived exosomes were 57.77 ± 43.82 pg/ml, 38.91 ± 14.12 pg/ml, and 32.53 ± 10.32 pg/ml in the J-Gout, J-HUA and oJIA groups, respectively (**Figure 7A**). The concentrations of serum-derived exosomal SERPIND1 were 10.26 ± 6.14 ng/ml, 8.21 ± 2.36 ng/ml, and 6.70 ± 1.85 ng/ml in the J-Gout, J-HUA and oJIA groups, respectively (**Figure 7B**). Both protein concentrations were highest in J-Gout and lowest in oJIA. Although the differences observed were not statistically significant, they were consistent with trends in proteomics.

**Figure 8:**
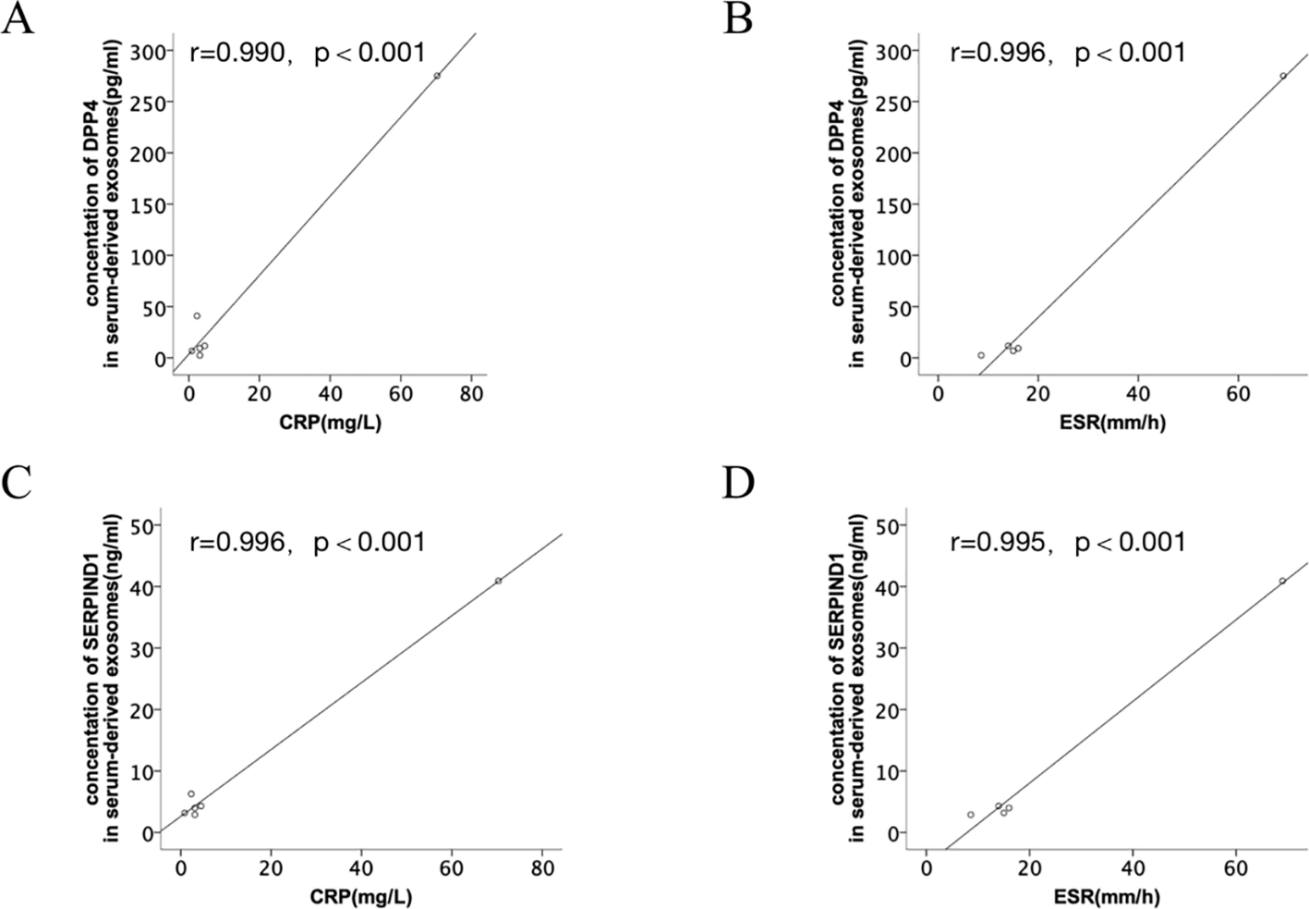
Correlation analysis between the differentially expressed proteins and clinical indicators. **(A, B)** Correlation analysis between DPP4 expression levels and clinical indicators (CRP and ESR). **(C, D)** Correlation analysis between SERPIND1 expression levels and clinical indicators (CRP and ESR).

The correlation between DPP4 and SERPIND1 expression levels and clinical indicators (CRP and ESR) was assessed. The results indicated that the DPP4 and SERPIND1 expression levels were positively correlated with CRP and ESR in serum-derived exosomes. The observed differences were statistically significant. (**Figure 8**). The expression levels of DPP4 and SERPIND1 did not correlate with age, sex, white blood cell count, neutrophil count, platelets, hemoglobin, serum uric acid level, RF and CCP. The results further verified that the differentially expressed proteins we selected were clinically significant.

## 4 Discussion

Exosomes can transfer bioactive lipids, nucleic acids, and proteins, to regulate gene expression and coordinate a broad spectrum of biological processes. Exosomes may become biomarkers for disease and potential candidates for disease therapy (16, 17). The advanced analytical approach of combining proteomics and bioinformatics analyses is currently used for discovering potential biomarkers for disease diagnosis and treatment. Because serum-derived exosomes are closely related to the pathogenesis of inflammation, there has been a recent increase in proteomic studies of exosomes for different rheumatic diseases (18, 19).

With changes in lifestyle, the proportion of J-Gout gradually increases, and there is a tendency for a younger age of onset, which can have a more significant impact on quality of life (20). The discussion of risk factors and clinical features is inadequate, and there are currently no guidelines for the management of J-Gout. JIA is an acquired autoinflammatory disease characterized by unexplained arthritis with onset before the age of 16 years, which can also present clinically with redness, pain, and limited mobility of the joints. The appearance of J-Gout is sometimes difficult to distinguish from other forms of JIA (21). Exosomes are essential mediators of intercellular communication and are involved in many processes. In our study, we used TMT proteomics technology to comprehensively analyze the protein composition of serum-derived exosomes in J-Gout, J-HUA and oJIA patients, and used bioinformatics to further explored the function of differentially abundant proteins in J-Gout.

The results showed that 88 differentially expressed proteins (13 upregulated and 75 downregulated) were found in J-Gout exosomes when compared with J-HUA exosomes. When compared with oJIA exosomes, 121 differentially expressed proteins (20 upregulated and 101 downregulated) were found in J-Gout exosomes. To comprehensively analyze the funcions of the differentially expressed proteins in J-Gout, 166 differentially expressed proteins were screened in J-Gout based on the combination of J-Gout vs. J-HUA and J-Gout vs. oJIA. The bioinformatics functional analysis of the differentially expressed proteins indicated that they were mainly enriched in “immune response”, “NF-kappa B signaling pathway”, “Fc epsilon RI signaling pathway” and “B cell receptor signaling pathway”. Previous studies revealed that macrophage phagocytosis of MSU is a key step in the pathogenesis of gout. MSU phagocytosis triggers NF-κB translocation to induce the expression and secretion of proinflammatory cytokines, such as IL-8, TNF-α and monocyte chemotactic protein-1 (MCP-1), which initiates the inflammatory response (22). By constructing murine models of gouty arthritis and observing joint swelling, synovial tissue edema, and inflammatory cell infiltration in mice, Cheng found that PAL attenuated MSU-induced gouty arthritis inflammation, indicating that Sirt1 alleviates M1 macrophage polarization and inflammation in gouty arthritis by inhibiting the MAPK/NF-κB/AP-1 pathway and activating the Nrf2/HO-1 pathway. Thus, activating Sirt1 may provide a new therapeutic target for gouty arthritis (23). In addition to its involvement in IgE-mediated antigen presentation, Fc ϵ RI also induces the transcription of cytokine genes by activating multiple signaling pathways. Fc epsilon RI signaling plays an important role in the pathogenesis of autoimmune allergic diseases, involving the activation of mast cells and the release of inflammatory mediators (24). NF-kappa B may be activated downstream of Fc epsilon RI signaling and thus participate in inflammatory responses. For example, mast cell activation may promote cytokine production and exacerbate inflammation through the NF-kappa B pathway. Fc epsilon RI-activated mast cells release cytokines such as TNF-α, which may further activate the inflammatory response through NF-kappa B and form a positive feedback loop. A study detected 256 unique extrachromosomal circular DNA elements (eccDNAs) in gout patients in the acute phase and found that these eccDNA genes were highly associated with immune and inflammatory responses, including the T-cell receptor, Fc ϵ RI and JAK-STAT signaling pathways (25). The hypothetical molecular mechanisms proposed above may provide therapeutic insights for J-Gout.

The uniquely expressed proteins in J-Gout were further screened based on the intersection of differentially expressed proteins in J-Gout vs. J-HUA and J-Gout vs. oJIA. The results showed that 6 proteins were uniquely highly expressed in J-Gout, of which SERPIND1 and DPP4 might be worthy of further study. ELISA results showed that the concentrations of SERPIND1 and DPP4 proteins in serum exosomes were highest in J-Gout and lowest in oJIA, which is consistent with the trend observed in the proteomics results. SERPIND1 is a thrombin inhibitor that restrains thrombin activity by interacting with heparin during the inflammatory response and affects the coagulation cascade. SERPIND1 can be cleaved by neutrophil elastase to promote neutrophil chemotaxis in acute inflammatory responses and also promote the release of leukocyte chemokines inducing those involved in angiogenesis. Guo’s study demonstrated that NF-κB could regulate SERPIND1 through the PI3K/AKT signaling pathway, thereby mediating cell migration, invasion, proliferation, apoptosis, and cell cycle regulation (26). Previous studies have shown that the pathogenesis of gout is closely related to the production of inflammatory factors in the acute inflammatory response. SERPIND1 acts as an inhibitor of thrombin and may reduce the release of inflammatory mediators by inhibiting thrombin. Thrombin can activate protease-activated receptors (PARs), thereby promoting the production of inflammatory factors, such as 1L-6 and IL-1β, while inhibition of thrombin may reduce the levels of these inflammatory factors and downregulate the expression of 1L-6 and IL-1β. In a gastric mucosal injury study, downregulation of SERPIND1 was associated with a decrease in inflammatory cytokines IL-6 and IL-1β, while expression of protective factors (eg, PGE2, SOD) was upregulated, suggesting that it may act through dual mechanisms (coagulation inhibition and anti-inflammation) (27). Alternatively, activation of the inflammasome often involves the action of coagulation factors and proteases. Thrombin activates the NLRP3 inflammasome, while SERPIND1 as a thrombin inhibitor may indirectly inhibit inflammasome activity by blocking this process, thereby reducing the release of pyroptosis-related factors (eg, caspase-1, IL-1β). DPP4 is expressed in many types of immune cells, and increasing research has focused on the potential role of DPP4 in autoimmune rheumatism. Both gout and diabetes mellitus type 2 (T2DM) are associated with HUA, and insulin resistance caused by HUA may be one of the causes involved in the pathogenesis. Previous studies showed that using antidiabetic agents reduced the risk of gout (28). Some novel antidiabetic agents, such as dipeptidyl peptidase-4 inhibition (DPP4I), reduced UA levels in patients with T2DM and had additional benefits for gout (29). These results indicated that DPP4 expression is closely related to the pathogenesis of gout. Our study also had an interesting finding that the DPP4 and SERPIND1 expression levels were positively correlated with CRP and ESR in serum-derived exosomes. Gout initially manifests as acute inflammatory arthritis, the activation of the NLRP3 inflammasome triggered by uric acid is considered a key pathogenic mechanism in the acute inflammatory response of gout, which leads to the production of proinflammatory cytokines, including interleukin-1β (IL-1β) and IL-18 (30, 31). Kim found that gout patients showed a higher expression of CXCL12 and proinflammatory cytokines, including IL-1β and IL-18, than members of the control group. Therefore, chemokine CXCL12 and its receptor CXCR4 might be considered to be potent therapeutic targets in uric acid-induced NLRP3 inflammasome activation in gout patients (32). Previous studies clarified that pharmacological inhibition of NLRP3 inflammasome assembly and activation may also be a promising approach for gouty arthritis treatment. Targeting NLRP3 through potentially effective drugs such as natural products, novel compounds, and non-coding RNAs (ncRNAs) for the treatment of mouse models of MSU-induced gouty arthritis may be important for the treatment of gouty arthritis (33). Therefore, SERPIND1 and DPP4 may participate in the occurrence and development of J-Gout.

There are some limitations in our study. Firstly, assessment of the basic demographic data of the subjects revealed significant differences in age and sex among the three groups. While the younger age of oJIA patients than J-Gout patients is consistent with the clinical characteristics of oJIA having a younger age of onset, the sample size was relatively small in our study. Secondly, because the study population was children, on the one hand due to ethical restrictions, and on the other hand, the small number of pediatric patients willing to enter the study resulted in the lack of healthy control group data in this study and the lack of female patients in the J-Gout group, although gout was more common in boys than in girls, we should further expand the sample size and conduct multicenter recruitment to obtain more objective results. Thirdly, the function of uniquely highly expressed proteins should be verified *in vivo* and *in vitro* to explore the significance of these proteins in greater depth.

In conclusion, the protein profiles of serum-derived exosomes in J-Gout were significantly different from those in J-HUA and oJIA. Some possible mechanisms were proposed. DPP4 and SERPIND1 were uniquely highly expressed in J-Gout. The highly expressed differential proteins in serum-derived exosomes are closely related to their function, which may be of great value in identifying potential biomarkers and further exploring the molecular mechanism of J-gout.

## Data Availability

The datasets presented in this study can be found in online repositories. The names of the repository/repositories and accession number(s) can be found in the article/supplementary material.
